# Treatment with immune checkpoint inhibitors after EGFR‐TKIs in *EGFR* ‐mutated lung cancer

**DOI:** 10.1111/1759-7714.14267

**Published:** 2021-12-13

**Authors:** Takashi Ito, Hiromi Nagashima, Masachika Akiyama, Yu Utsumi, Hideomi Sato, Shinji Chiba, Mayu Sugai, Kenji Ube, Yoshiaki Mori, Kana Watanabe, Tatsuro Fukuhara, Makoto Maemondo

**Affiliations:** ^1^ Division of Pulmonary Medicine, Department of Internal Medicine Iwate Medical University School of Medicine Yahaba Japan; ^2^ Department of Respiratory Medicine Iwate Prefectural Central Hospital Morioka Japan; ^3^ Department of Respiratory Medicine Miyagi Cancer Center Natori Japan

**Keywords:** *EGFR* mutation, EGFR tyrosine kinase inhibitor, immune checkpoint inhibitor, L858R mutation, non‐small cell lung cancer

## Abstract

**Background:**

Epidermal growth factor receptor‐tyrosine kinase inhibitors (EGFR‐TKIs) have become the gold standard for *EGFR*‐mutated non‐small cell lung cancer (NSCLC) treatment. Immune checkpoint inhibitors (ICIs) have been developed for the treatment of several malignancies, including lung cancer. However, it is known that ICIs have poorer efficacy in *EGFR*‐mutated NSCLC.

**Methods:**

We collected data for patients with *EGFR*‐mutated NSCLC receiving monotherapy with ICIs after EGFR‐TKIs between December 2015 and March 2020 in three institutions, and retrospectively analyzed the association between patient characteristics and efficacy of ICIs.

**Results:**

A total of 25 patients were included in this study. We defined responders as patients undergoing 90 days or longer of ICI treatment. Comparing characteristics between responders and non‐responders, more tumors with L858R *EGFR* mutation were observed in responders than in non‐responders (L858R: 66.7% and 25.0%, respectively, *p* < 0.05). There was no difference in incidence of T790M resistance mutation before ICI treatment. The PD‐L1 positive rate was slightly higher in responders but not statistically significant (22.2% and 12.5%, respectively). Median duration of EGFR‐TKI pretreatment was shorter in ICI responders compared with nonresponders (13.3 and 19.9 months, respectively). The survival of patients with L858R tumors was significantly longer than that of patients with exon 19 deletion (HR: 0.35, 95% CI: 0.13–0.93, *p* = 0.026).

**Conclusions:**

ICI treatment tends to have better efficacy in patients with L858R‐mutated tumors. This study suggests that patients with L858R‐mutated NSCLC are candidates for ICI treatment after EGFR‐TKI treatment.

## Graphical abstract

Efficacy of ICI in a part of patients with *EGFR*‐mutated NSCLC was observed. ICI treatments in tumors with L858R mutation achieved a better response than exon 19 deletion. This study suggests that patients with L858R‐mutated NSCLC are candidates for ICI treatment after EGFR‐TKI treatment.

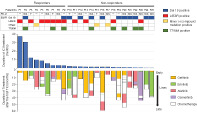

## INTRODUCTION

The epidermal growth factor receptor (EGFR) gene mutation is the most frequent driver mutation to strongly promote cancer progression in non‐small cell lung cancer (NSCLC). There are significant differences in the frequency of *EGFR* mutations, with about 10% in cases of lung adenocarcinoma in Caucasians, but about 50% in cases of lung adenocarcinoma in East Asians.^1^, ^2^ Developing effective therapeutic strategies for *EGFR* mutation‐positive NSCLC is one issue in NSCLC treatment. Several phase III trials of EGFR‐tyrosine kinase inhibitors (TKIs), targeting therapy for *EGFR* gene mutations, and showed significant efficacy for EGFR‐TKIs in EGFR‐mutated tumors compared with platinum combination chemotherapy.^3^, ^4^ EGFR‐TKIs have become the standard first‐line treatment for *EGFR* mutation‐positive NSCLC.^5^ Various subtypes of EGFR gene mutations have been observed, but the most common genetic alterations are exon 19 deletion and exon 21 L858R point mutations, which together account for approximately 80% of EGFR mutations, and so‐called activating mutation. EGFR‐TKIs appear highly responsive to activating *EGFR*‐mutated tumors.^6^, ^7^ However, one clinical problem is that acquired resistance mutations occur about one year after administration of EGFR‐TKIs. T790M mutations caused by first‐ and second‐generation EGFR‐TKIs (gefitinib, erlotinib, and afatinib) are known gatekeeper mutations and account for 40% to 50% of resistance mechanisms to first‐ and second‐generation EGFR‐TKI.^8^ Osimertinib, a third‐generation EGFR‐TKI, has a high affinity for EGFR with both T790M mutation and activating mutation. A phase III study of osimertinib in a first‐line setting showed a higher effect on activating EGFR mutation compared with first‐generation EGFR‐TKI. Osimertinib has become a standard first‐line therapy for NSCLC with *EGFR* activating mutation. However, the next most optimal regimen for NSCLC patients without T790M mutations after failure of first and second generation EGFR‐TKIs or for patients who experienced failure with osimertinib has been poorly validated, and platinum combination chemotherapy is often selected. We consider post‐osimertinib treatments as an unresolved issue in treatment strategies for EGFR‐mutated NSCLC.

In recent years, the effectiveness of immune‐checkpoint inhibitor (ICI) therapy has been proven in NSCLC as well as in various cancer types, and it is widely used as a key treatment strategy for cancers.^9^ However, in EGFR mutation‐positive NSCLC, the efficacy of ICI treatment has not been verified, and no clinical benefits for most EGFR‐mutated tumors have been shown.

ICI treatment has been approved for lung cancer, but it has been reported that ICI treatments are less beneficial in *EGFR*‐mutated NSCLC, and patients with *EGFR*‐mutated tumors have been excluded from most clinical studies. In terms of EGFR‐wild NSCLC, predictive factors of the effect of ICI including PD‐L1 status and smoking status have been evaluated. One report suggested that the response to first‐line platinum combination chemotherapy may be a predictor of the subsequent ICI effect in EGFR mutation‐negative NSCLC.^10^ The relationship between the effect of pretreatment and the effect of ICI is one potential predictor of ICI efficacy. Recently, it has been demonstrated that gene alterations including TP53 mutations or genomic alterations, tumor mutation burden (TMB), and the fraction of copy number‐altered genome, can also affect the response to immunotherapy.^11^, ^12^ Next‐generation sequencing (NGS) is a method for comprehensively analyzing gene alterations, and is attracting attention as a method for searching for predictors of ICI effects in lung cancer patients.^13^

In this study, we retrospectively analyzed the efficacy of ICI in patients with *EGFR* gene mutation‐positive lung cancer who received ICI treatment after treatment with EGFR‐TKIs and evaluated the relationship between ICI treatment period and characteristics including EGFR subtype and treatment period of previous EGFR‐TKIs.

## METHODS

### Patients and data collection

From December 2015 to March 2020, 25 cases meeting the registration criteria were collected from three hospitals: Iwate Medical University Hospital, Iwate Prefectural Central Hospital, and Miyagi Cancer Center. The main eligibility criteria were unresectable or postoperatively recurrent NSCLC harboring *EGFR* mutation‐positive; previous treatment with gefitinib, erlotinib, afatinib, or osimertinib; and monotherapy with nivolumab, pembrolizumab, or atezolizumab after the EGFR‐TKI treatment. ICI did not have to be administered after EGFR‐TKIs sequentially, and chemotherapies were allowed between EGFR‐TKI and ICI. Cases receiving combination therapy with ICI and chemotherapy and cases with a history of autoimmune disease, interstitial pneumonia, or symptomatic cerebrovascular disease were excluded.

For 25 patients who met the predetermined eligibility criteria, clinical data were collected from electronic medical records. Clinical data included gender, age, smoking history, stage at diagnosis, histological type, metastatic lesion, type of *EGFR* mutation, type of TKI, duration of TKI administration, status of T790M mutation, PD‐L1 expression rate, and ICI reactivity (type of ICI, duration of ICI administration, and adverse events). Tumor evaluation of CT and MRI images was performed according to Response Evaluation Criteria in Solid Tumors (RECIST) v1.1. Toxicity was assessed using the Common Terminology Criteria for Adverse Events (CTCAE) v4.0. The relationship between the ICI treatment actually received and the tumor evaluation data was carefully checked using the case report form, medical record, CT and MRI for assessment of pseudoprogression and treatment beyond PD. This study was conducted according to the protocol approved by the ethics committee established at each hospital (approval No. MH2019‐155 at Iwate Medical University).

### Sample collection and next‐generation sequencing

Of the cases in which medical biopsy or surgery was performed within the past five years, 12 specimens with enough formalin‐fixed paraffin‐embedded (FFPE) tissue samples for NGS were selected. A 5 μm‐thick‐sliced slide was prepared. NGS was performed on six samples, excluding six samples with poor quality extracted DNA. A DNA panel including 407 genes on drug targets, prognostic and diagnostic markers were used (Table S1). Nonsynonymous single nucleotide variants (SNVs), small insertions and deletions (indels), copy number variants (CNVs), microsatellite instability (MSI), and TMB were detected using CancerSCAN v3. DNA extraction from samples, DNA quality evaluation, and NGS analysis were all carried out by Geninus Inc. (Seoul, Korea), which was commissioned as a research cooperation facility. Details of the method are shown in the report by Shin et al.^14^

### Statistical analysis

The duration of treatment with EGFR‐TKIs and ICIs was defined as the period from the start date of administration of each drug to the date of termination of administration due to disease progression or death. Patients receiving ICI continuously were censored on the last observation date. The overall survival (OS) of ICI was defined as the period from the start date of ICI administration to the date of death, and the survivors were considered as censored cases at the last observation date. Characteristics were compared between two groups, one with ICI treatment for less than 90 days and the other with ICI treatment for 90 days or more. The classification variable was tested using Fisher's exact test or the Chi‐square test, and the continuous variable was tested using the Mann–Whitney test. Regarding the subtype of *EGFR* mutation, the objective response rate (ORR) and the disease control rate (DCR) were compared based on the best response of ICI between the two groups of exon 19 deletions and L858R. The duration of ICI treatment and OS survival curve were computed using the Kaplan–Meier method, and significant differences were verified using the log‐rank test. Univariate analysis of ICI treatment duration in a forest plot was evaluated with the log‐rank test. Hazard ratio (HR) and 95% confidence interval (CI) were calculated using Cox's proportional hazards model. *p*‐values < 0.05 were considered statistically significant. For statistical analysis, SPSS statistics version 25 (IBM) and EZR (Saitama Medical Center, Jichi Medical University, Saitama, Japan) were used.

## RESULTS

### Patient characteristics

The patient characteristics are shown in Table 1. There were 15 men (60%) with a median age of 67 years (38–80 years). Eleven patients (44%) had a history of smoking, and 10 patients (40%) had postoperative recurrence. The histological type at diagnosis was adenocarcinoma in all cases. The ICI monotherapies after EGFR‐TKI administration were nivolumab in 21 cases, pembrolizumab in two cases, and atezolizumab in two cases. Of the 24 cases excluding de novo T790M, the resistance T790M mutation was detected by biopsy before the start of ICIs in seven cases (29.2%), and 14 cases (58.3%) were T790M negative. The T790M was not verified in three cases. PD‐L1 tumor proportion score (TPS) before ICI administration was less than 1% in eight cases (32%), 1% or more in four cases (16%), and in the remaining 13 cases it was not evaluated.

**TABLE 1:** Patient characteristics

	Number of patients (%)			*p*‐value
	Total (%) (*n* = 25)	Nonresponder (*n* = 16)	Responder (*n* = 9)	
Gender				1.000
Male	15 (60.0)	10 (62.5)	5 (55.6)	
Female	10 (40.0)	6 (37.5)	4 (44.4)	
Median age (range), years	67 (38–80)	66 (38–80)	71 (40–77)	0.276
Smoking history				0.688
Current or former	11 (44.0)	7 (43.8)	5 (55.6)	
Never	14 (56.0)	9 (56.2)	4 (44.4)	
Stage				0.691
IIIB–IV	15 (60.0)	9 (56.3)	6 (66.7)	
Recurrence	10 (40.0)	7 (43.7)	3 (33.3)	
Histology				
Adenocarcinoma	25 (100)	16 (100)	9 (100)	
Others	0			
Metastasis	23 (92.0)	16 (100)	7 (77.8)	0.120
CNS	7 (30.4)	4 (25.0)	3 (42.9)	0.673
Others	16 (69.6)	12 (75.0)	4 (57.1)	
*EGFR* mutation status				0.043
Del 19	11 (44.0)	10 (62.5)	1 (11.1)	
L858R	10 (40.0)	4 (25.0)	6 (66.7)	
Uncommon or compound	4 (16.0)	2 (12.5)	2 (22.2)	
T790M				0.371
Positive	8 (32.0)	5 (31.3)	3 (33.3)	
Negative	14 (56.0)	8 (50.0)	6 (66.7)	
Unknown	3 (12.0)	3 (18.7)	0	
ICIs				0.513
Nivolumab	21 (84.0)	13 (81.3)	8 (88.9)	
Pembrolizumab	2 (8.0)	1 (6.3)	1 (11.1)	
Atezolizumab	2 (8.0)	2 (12.5)	0	
PD‐L1 expression				1.000
<1%	8 (32.0)	4 (25.0)	4 (44.4)	
≧1%	4 (16.0)	2 (12.5)	2 (22.2)	
Not available	13 (52.0)	10 (62.5)	3 (33.3)	
Number of EGFR‐TKI regimens				0.115
1	11 (44.0)	5 (31.3)	6 (66.7)	
≧2	14 (56.0)	11 (68.7)	3 (33.3)	
ICI line				0.493
Second	1 (4.0)	0	1 (11.1)	
Third	9 (36.0)	5 (31.3)	4 (44.4)	
≧Fourth	13 (52.0)	9 (60.0)	4 (44.4)	
Agents immediately prior to ICI treatment				1.000
EGFR‐TKI	12 (48.0)	8 (50.0)	4 (44.4)	
Chemotherapy	13 (52.0)	8 (50.0)	5 (55.6)	
Duration of all EGFR‐TKI treatments, median months (range)	17.0 (2.7–45.2)	19.9 (2.7–45.2)	13.3 (2.8–34.1)	0.251
Period between termination of EGFR‐TKI and initiation of ICI, median months (range)	4.5 (0.03–22.8)	2.3 (0.03–15.6)	5.8 (0.4–22.8)	0.257

Abbreviations: CNS, central nervous system; Del 19, exon 19 deletion; EGFR‐TKI, epidermal growth factor receptor‐tyrosine kinase inhibitor; ICIs, immune checkpoint inhibitors; PD‐L1, programmed death ligand 1.

The median duration of ICI treatment was 1.4 months. All patients were divided into two groups by treatment duration, with nonresponder or responder defined by duration of ICI treatment of less than 90 days, or 90 days or more, respectively. Men accounted for 62.5% and 55.6% of nonresponders and responders, respectively, and median age was 66 and 71 years, rate of smoking history was 43.8% and 55.6%, and postoperative recurrence rate was 43.7% and 33.3% in nonresponders and responders, respectively. In nonresponders and responders, EGFR gene mutation was 62.5% and 11.1% for exon 19 deletion, and 25.0% and 66.7% for L858R, T790M positive rates were 31.3% and 33.3%, the rates of PD‐L1 positive defined as TPS 1% or higher were 12.5% and 22.2%, percentages receiving multiple regimens of EGFR‐TKI treatment were 68.7% and 33.3%, and median duration of TKI treatment was 19.9 and 13.3 months, respectively. The proportion of patients receiving chemotherapy immediately prior to ICI treatment was 50.0% for nonresponders and 55.6% for responders. In addition, the median period between termination of EGFR‐TKI treatment and initiation of ICI treatment was 2.3 months for nonresponders and 5.8 months for responders. Among these factors, significantly more L858R mutations were observed in responders (*p* < 0.05). As for other subtypes of EGFR, uncommon mutations were found in G719X, and exon 20 insertion and compound mutations were found in L858R and de novo T790M, and exon 19 deletion and L858R, and their distribution between two groups is shown in Table 1. In addition, two cases in the responder group showed transformation into squamous cell carcinoma revealed by rebiopsies during the treatment course, and the EGFR subtype in both cases was L858R mutation. There were no cases beyond PD and no cases of pseudoprogression.

### Best response

The best responses during ICI treatment are shown in Table 2. There was one (4%) complete response (CR), four (16%) partial responses (PR), four (16%) with stable disease (SD), and 16 (64%) with progressive disease (PD) observed. ORR was 20% and DCR was 36%. Comparing EGFR subtype mutation, ORR was 9.1% and 30.0%, and DCR was 27.3% and 50.0%, respectively, in exon 19 deletions and L858R. ICIs tended to have a higher antitumor effect on L858R tumors. In four cases of uncommon mutation or compound mutation, one (25%) PR case and three (75%) PD cases were observed. In addition, there was no difference in response rate between PD‐L1 positive and PD‐L1 negative patients. Interestingly, a higher response rate was observed in non‐smokers.

**TABLE 2:** Patient characteristics

	Total (*n* = 25)	EGFR subtypes			Smoking history		PD‐L1	
		Del 19 (*n* = 11)	L858R (*n* = 10)	Others^a^ (*n* = 4)	– (*n* = 13)	+ (*n* = 12)	– (*n* = 8)	+ (*n* = 4)
CR	1 (4)	1 (9.1)	0	0	1 (7.7)	0	0	0
PR	4 (16)	0	3 (30)	1 (25)	2 (15.4)	2 (16.6)	2 (25)	1 (25)
SD	4 (16)	2 (18.2)	2 (20)	0	2 (15.4)	2 (16.6)	2 (25)	0
PD	16 (64)	8 (72.7)	5 (50)	3 (75)	8 (61.5)	8 (66.7)	4 (50)	3 (75)
ORR (%)	20	9.1	30	20	23.1	16.6	25	25
DCR (%)	36	27.3	50	20	38.5	33.3	50	25

Abbreviations: CR, complete response; DCR, disease control rate; EGFR, epidermal growth factor receptor; ORR, objective response rate; PD, progressive disease; PD‐L1, programmed death ligand 1; PR, partial response; SD, stable disease.

^a^ G719X, G719A, ex20 insertion, and exon 19 deletion + L858R.

### Duration of treatment

**FIGURE 1:**
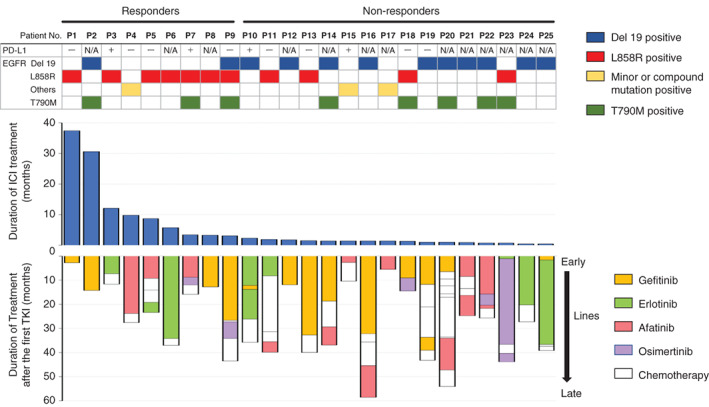
Treatment period of EGFR‐TKI and ICI in each of the 25 cases. The upper bar shows the duration of ICI treatment, and the lower bar shows the duration of treatment from the time of the first EGFR‐TKI treatment. PD‐L1, programmed death ligand 1; N/A, not available; ICI, immune checkpoint inhibitor; TKI, tyrosine kinase inhibitor; Del 19, exon 19 deletion; minor, minor mutation

Each treatment period of EGFR‐TKI and ICI for all 25 cases is shown in Figure 1. Patients with L858R‐mutated tumors had a relatively longer treatment period of ICI compared with exon 19 deletions. In addition, ICI treatments continued longer in patients with shorter previous TKI treatments. Interestingly, the patient with the longest ICI treatment period had L858R tumors and experienced the shortest treatment period of EGFR‐TKI. In the cases involving tumors with uncommon mutations and compound mutations, no relationship between treatment period of EGFR‐TKI and efficacy of ICI was observed.

### Univariate analysis of ICI treatment periods

**FIGURE 2:**
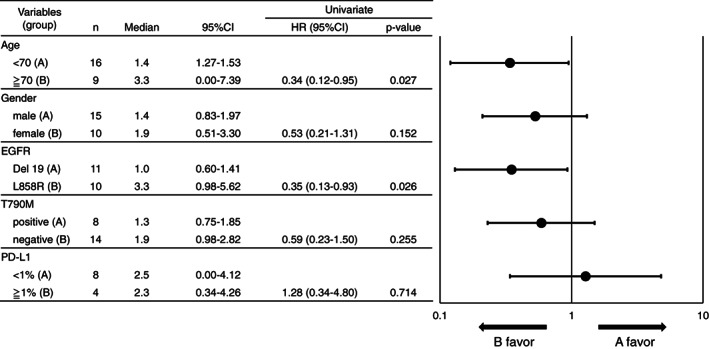
Univariate analysis and Forest plot for duration of ICI treatment by subgroups. Main characteristics including EGFR, T790M, PD‐L1, gender, and age were evaluated. The *p*‐value and hazard ratio were determined using the log‐rank test and Cox's proportional hazards model, respectively. HR, hazard ratio; CI, confidence interval; EGFR, epidermal growth factor receptor; PD‐L1, programmed death ligand 1

**FIGURE 3:**
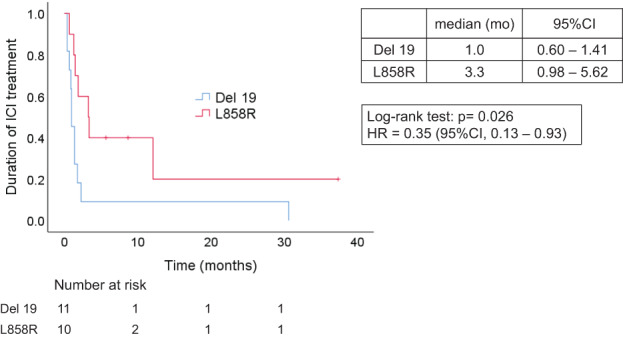
Kaplan–Meier curves for duration of ICI treatment according to EGFR subtypes (exon 19 deletion versus L858R). Duration of ICI treatment for patients with L858R was significantly longer compared with exon 19 deletion (median 3.3 vs. 1.0 months; HR, 0.35; 95% CI: 0.13–0.93; log‐rank test *p* = 0.026). Del 19, exon 19 deletion; HR, hazard ratio; CI, confidence interval

Univariate analysis of duration of ICI treatment and the main characteristics including gender, age, EGFR subtypes, T790M mutations, and PD‐L1 expression was performed (Figure 2). For the EGFR subtypes, the median duration of ICI treatment was 1.0 months (95% CI: 0.60–1.41) in the exon 19 deletion group, and 3.3 months (95% CI: 0.98–5.62) in the L858 group (HR 0.35, 95% CI: 0.13–0.93, *p* = 0.026). Therefore, duration of ICI treatment was significantly longer for the L858R group than for the exon 19 deletion groups. Kaplan–Meier analysis of duration of ICI treatment in EGFR subtypes is shown (Figure 3). Similarly, patients aged 70 years and older had a significantly longer ICI treatment period than those aged under 70 years (HR 0.34, 95% CI: 0.12–0.95, *p* = 0.027). There were no statistically significant differences in gender or T790M subgroups ([HR 0.53, 95% CI: 0.21–1.31, *p* = 0.152], [HR = 0.59, 95% CI: 0.23–1.50, *p* = 0.255], respectively).

### OS of ICI treatment

Kaplan–Meier analysis of OS from the start of ICI revealed that the medians of OS for exon 19 deletion and L858R were 15.7 months (95% CI: 8.76–22.64) and 14.8 months (95% CI: 0.00–34.61), respectively (HR 1.00, 95% CI: 0.29–3.47, *p* = 0.997) (Figure S1). Understandably, when OS was compared between ICI responders and nonresponders, responders achieved a longer OS (Figure S1).

### Safety

In this study, adverse events were observed during ICI administration in eight cases (32%), but no serious adverse events due to ICI requiring discontinuation of treatment were observed (Table S2). All adverse events including immune related adverse events were grade 2 or lower, and no patient experienced toxicities of ICI requiring hospitalization. Although it is a concern that ICI treatment after EGFR‐TKI treatment frequently induces interstitial lung disease (ILD), there was no ILD in this study.

### Next‐generation sequencing

In total six cases (P2, P4, P8, P18, P22, P22, P24) were analyzed using NGS (Figure S3). As a gene that generated SNVs and Indels, TERT was detected in all six cases. In addition, TP53 variants were detected in three cases (50%), all of which were nonresponder cases. Detection of CNVs of CDKN2A and FOXL2 in three cases was confirmed. In all six cases, MSI was microsatellite‐stable (MSS) and TMB was low.

## DISCUSSION

This was a retrospective study evaluating ICI treatment after failure of EGFR‐TKI in *EGFR*‐mutated NSCLC. Although ICI treatment in *EGFR*‐mutated NSCLC has been considered to have poor efficacy, efficacy tends to be better in patients with tumor L858R mutation.

The efficacy of ICI as second‐line treatment after EGFR‐TKI for *EGFR* mutation‐positive NSCLC has not been fully validated, and EGFR‐positive NSCLC has been ruled out in many clinical studies of ICI. Some reports have shown that ICI treatment is less effective for EGFR‐positive NSCLC.^15^, ^16^ In a meta‐analysis of second‐line NSCLC phase II and III trials, ICI treatment benefited OS over docetaxel in EGFR mutation‐positive cases (HR 1.11, 95% CI: 0.80–1.53, *p* = 0.54) (not shown). The benefits for OS have been shown across the population, but have not been observed in *EGFR* mutation‐positive cases.^17^ In a single‐center report by Gainor et al., the ORR of ICI in EGFR mutation‐positive or ALK fusion gene‐positive NSCLC was as low as 3.6%.^18^ Mazieres et al. reported that a study of ICI in *EGFR* mutant NSCLC regardless of receiving EGFR‐TKI treatment showed 2.1 months mPFS, ORR 12.2%, and DCR 33.1%.^19^ Our current study showed 20% for ORR and 36% for DCR. The ORR for ICI in this study was comparable with the 19% ORR in the Checkmate 057 study comparing nivolumab and docetaxel in nonsquamous NSCLC.^9^ The higher response rate in this study derived from the high response rate of L858R tumors (RR 30% in L858R, RR 9.1% in ex 19 Del).

It has been pointed out that the EGFR subtype is an important factor associated with the therapeutic response of EGFR‐TKIs, and that L858R tends to be slightly less effective than exon 19 deletion in EGFR‐TKIs.^20^ L858R frequently causes compound mutations, which may be related to the fact that tumor mutation burden (TMB) is a strong independent predictive factor.^13^, ^21^ Exon 19 deletion EGFR does not need dimerization to activate downstream signals, but L858R EGFR needs dimerization to transduce downstream signals. This molecular biological difference between exon 19 deletions and L858R affects rapid tumor progression and sensitivity to EGFR‐TKI.^22^ Our study showed that L858R responded significantly to ICI compared with exon 19 deletions. This is consistent with a previous report.^23^ In considering strategies for *EGFR*‐mutated NSCLC it is vital to recognize that the EGFR subtype shows the opposite responsiveness for EGFR‐TKIs compared with ICI treatment.

In this study, NGS was performed in only six cases with appropriate tissues for NGS, so it is difficult to find gene alterations related to the effectiveness of ICI. However, some interesting results were obtained. TERT, in which mutations were detected in all six cases, encodes telomerase reverse transcriptase, which is a major factor in telomerase reactivation and accelerates tumor progression. Mutations in the TERT promoter are considered poor prognostic factors for NSCLC.^24^ TP53 is one of the most important tumor suppressor genes. TP53 mutation in lung adenocarcinoma is known as a worse prognostic factor for survival.^25^ Assoun et al. reported that ICI treatments in TP53‐mutated NSCLC showed longer PFS and OS compared with TP53 wild‐type.^26^ In this study, all cases with TP53 mutation belonged to nonresponder. Another report showed that TP53 mutations in plasma negatively affected OS both in patients treated with or without ICI and KRAS/STK11/TP53 comutation affected OS only in patients treated with ICI.^27^ Studies with more cases need to verify genetic mechanisms of efficacy of ICI after EGFR‐TKI treatments.

In this study, no patients experienced ILD despite ICI treatment after EGFR‐TKI treatment. All adverse events of ICI treatment were mild and manageable. We believe that the safety results of this study provide important findings for therapeutic strategies that use ICIs after treatment with EGFR‐TKI.

This study has some limitations. First, it was a retrospective study, and unlike a prospective study, imaging tests were performed according to the clinical situation. As a result, the treatment period was used instead of PFS to evaluate the effect of ICI treatment. Second, because of insufficient description in the medical records, we could not collect other factors including performance status that may be related to the effects of ICI treatment. Third, due to the small number of cases, it was difficult to analyze independent factors with statistically significant differences using multivariate analysis. However, it was possible to find factors related to the effect of ICI treatment. In our study, validation with ICI alone suggested that differences in genetic background may affect the efficacy of ICI. To elucidate whether the same tendency is observed in the combination therapy of ICI and chemotherapy, future studies are warranted.

In conclusion, ICI treatment for *EGFR*‐mutated NSCLC is not necessarily off label, but it is vital to select appropriate patients for ICI treatment. From the results of the current study, we recommend patients with L858R‐mutated NSCLC as candidates for ICI treatment after EGFR‐TKI treatment.

## CONFLICT OF INTEREST

Makoto Maemondo received lecture fees from Ono, Bristol‐Meier, Chugai, AstraZeneca, and Boehringer‐Ingelheim. He also conducted a clinical study supported by Chugai. The other authors have no conflict of interest to declare.

## Supporting information


**Figure S1.** Kaplan–Meier curves of OS from the start of ICI in exon 19 deletions and L858R.
**Figure S2.** Kaplan–Meier curves of duration of ICI treatment in non‐responders and responders.
**Figure S3.** NGS analysis of six cases.

**Table S1.** DNA panel used in next‐generation sequencing
**Table S2.** Adverse events.
