# Supplementary material for: Treatment with immune checkpoint inhibitors after EGFR‐TKIs in EGFR ‐mutated lung cancer
Source: Thorac Cancer. 2021 Dec 13;13(3):386–93. doi: 10.1111/1759-7714.14267 (PMC8807326; doi:10.1111/1759-7714.14267)
Supplement: Supplementary file 1 — Figure S1. Kaplan–Meier curves of OS from the start of ICI in exon 19 deletions and L858R. Figure S2. Kaplan–Meier curves of duration of ICI treatment in non‐responders and responders. Figure S3. NGS analysis of six cases. [file TCA-13-386-s002.pptx]

## Slide 1
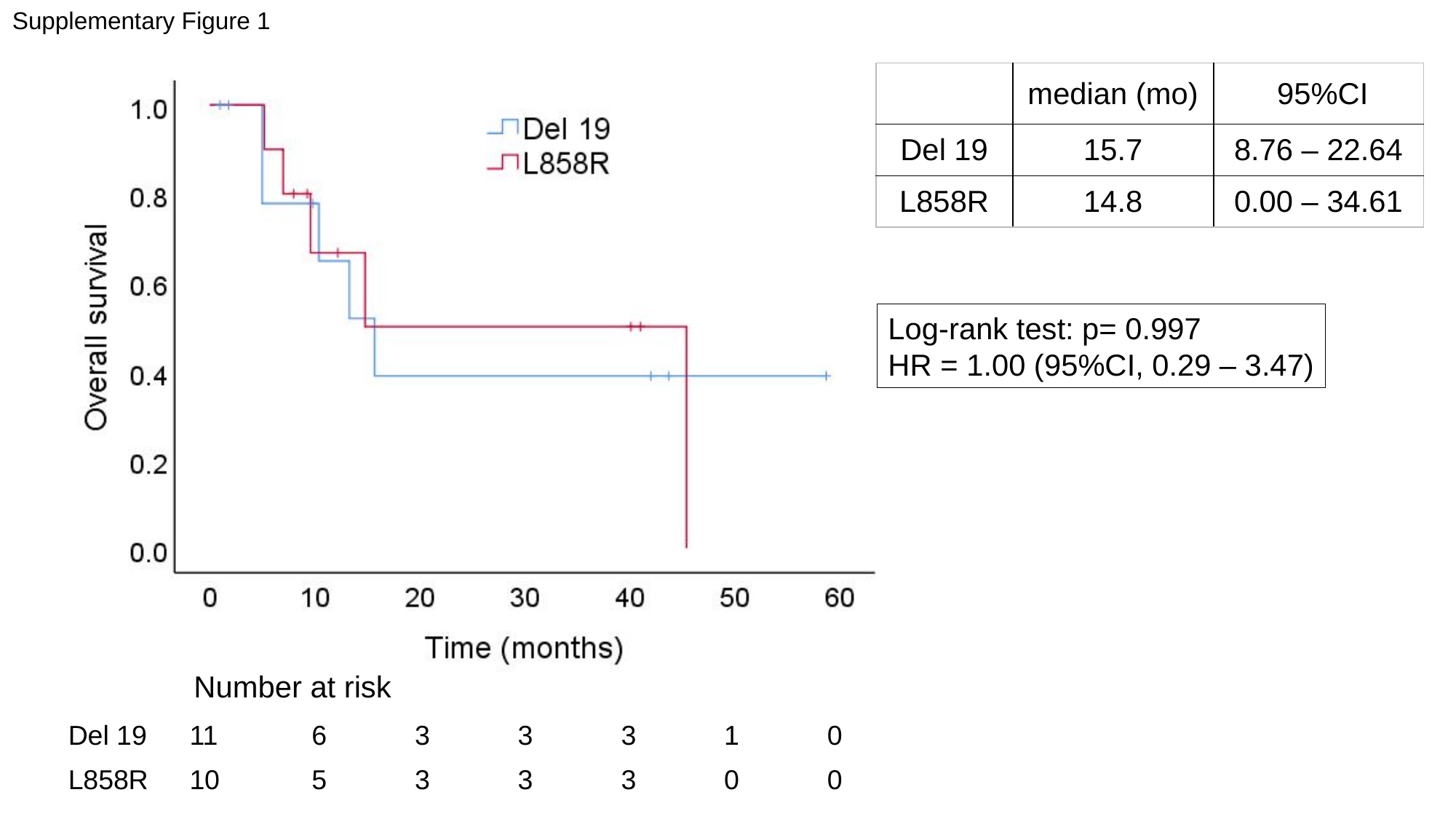

Supplementary Figure 1
| | median (mo) | 95%CI |
| --- | --- | --- |
| Del 19 | 15.7 | 8.76 – 22.64 |
| L858R | 14.8 | 0.00 – 34.61 |
Log-rank test: p= 0.997
HR = 1.00 (95%CI, 0.29 – 3.47)
Number at risk
| Del 19 | 11 | 6 | 3 | 3 | 3 | 1 | 0 |
| --- | --- | --- | --- | --- | --- | --- | --- |
| L858R | 10 | 5 | 3 | 3 | 3 | 0 | 0 |

## Slide 2
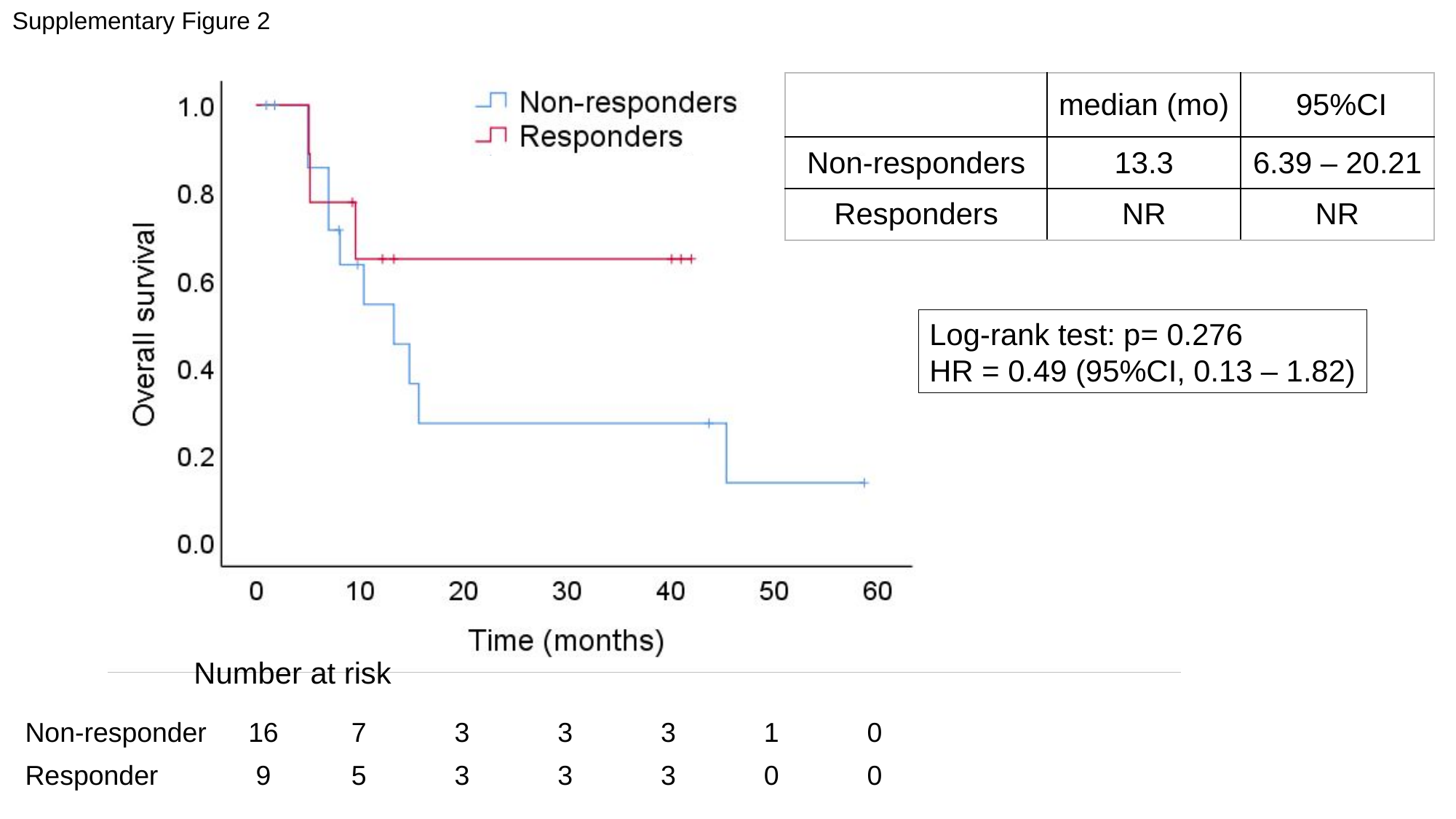

Supplementary Figure 2
| | median (mo) | 95%CI |
| --- | --- | --- |
| Non-responders | 13.3 | 6.39 – 20.21 |
| Responders | NR | NR |
Log-rank test: p= 0.276
HR = 0.49 (95%CI, 0.13 – 1.82)
Number at risk
| Non-responder | 16 | 7 | 3 | 3 | 3 | 1 | 0 |
| --- | --- | --- | --- | --- | --- | --- | --- |
| Responder | 9 | 5 | 3 | 3 | 3 | 0 | 0 |

## Slide 3
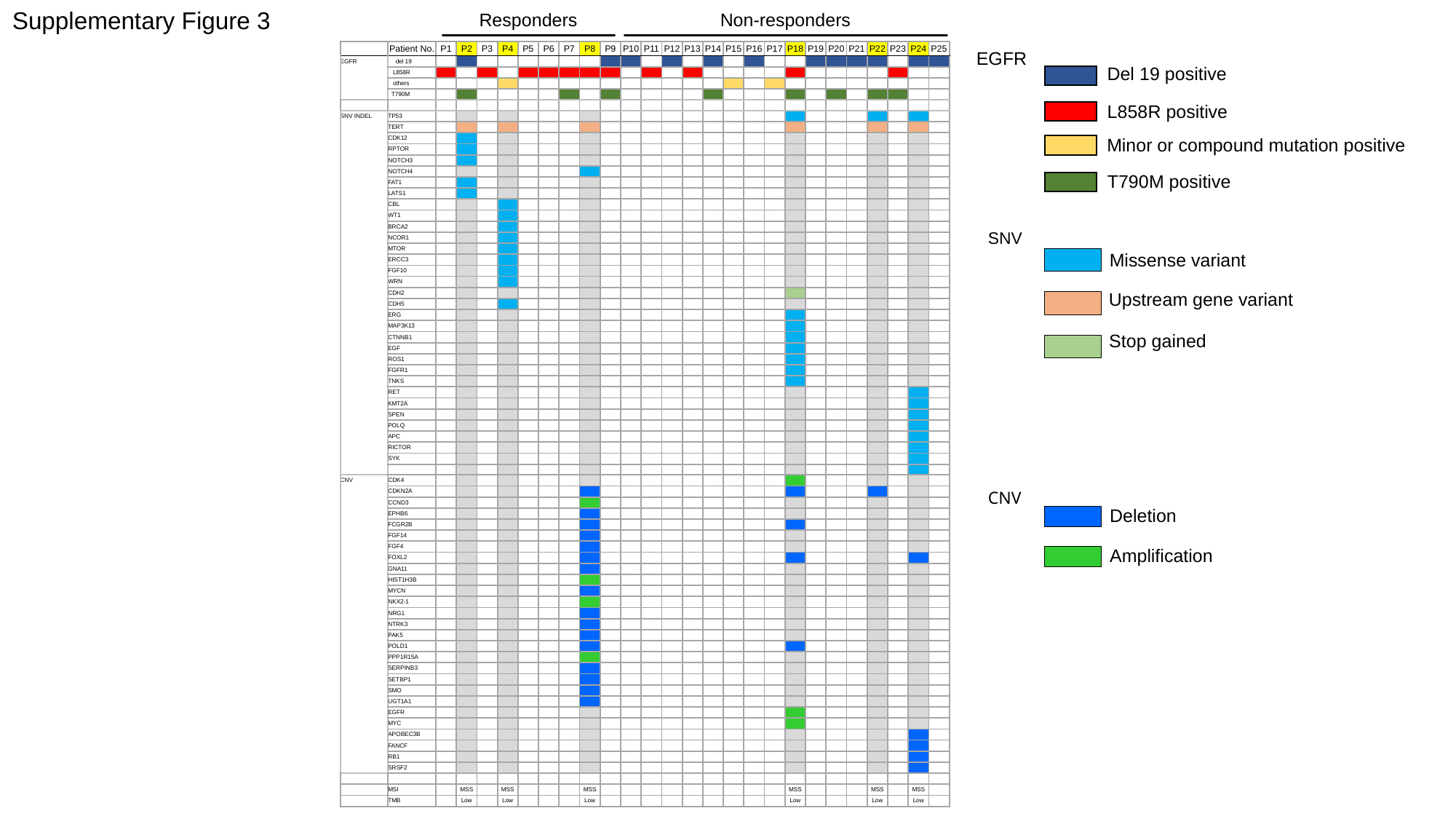

Supplementary Figure 3
Responders
Non-responders
| | Patient No. | P1 | P2 | P3 | P4 | P5 | P6 | P7 | P8 | P9 | P10 | P11 | P12 | P13 | P14 | P15 | P16 | P17 | P18 | P19 | P20 | P21 | P22 | P23 | P24 | P25 |
| --- | --- | --- | --- | --- | --- | --- | --- | --- | --- | --- | --- | --- | --- | --- | --- | --- | --- | --- | --- | --- | --- | --- | --- | --- | --- | --- |
| EGFR | del 19 | | | | | | | | | | | | | | | | | | | | | | | | | |
| | L858R | | | | | | | | | | | | | | | | | | | | | | | | | |
| | others | | | | | | | | | | | | | | | | | | | | | | | | | |
| | T790M | | | | | | | | | | | | | | | | | | | | | | | | | |
| | | | | | | | | | | | | | | | | | | | | | | | | | | |
| SNV INDEL | TP53 | | | | | | | | | | | | | | | | | | | | | | | | | |
| | TERT | | | | | | | | | | | | | | | | | | | | | | | | | |
| | CDK12 | | | | | | | | | | | | | | | | | | | | | | | | | |
| | RPTOR | | | | | | | | | | | | | | | | | | | | | | | | | |
| | NOTCH3 | | | | | | | | | | | | | | | | | | | | | | | | | |
| | NOTCH4 | | | | | | | | | | | | | | | | | | | | | | | | | |
| | FAT1 | | | | | | | | | | | | | | | | | | | | | | | | | |
| | LATS1 | | | | | | | | | | | | | | | | | | | | | | | | | |
| | CBL | | | | | | | | | | | | | | | | | | | | | | | | | |
| | WT1 | | | | | | | | | | | | | | | | | | | | | | | | | |
| | BRCA2 | | | | | | | | | | | | | | | | | | | | | | | | | |
| | NCOR1 | | | | | | | | | | | | | | | | | | | | | | | | | |
| | MTOR | | | | | | | | | | | | | | | | | | | | | | | | | |
| | ERCC3 | | | | | | | | | | | | | | | | | | | | | | | | | |
| | FGF10 | | | | | | | | | | | | | | | | | | | | | | | | | |
| | WRN | | | | | | | | | | | | | | | | | | | | | | | | | |
| | CDH2 | | | | | | | | | | | | | | | | | | | | | | | | | |
| | CDH5 | | | | | | | | | | | | | | | | | | | | | | | | | |
| | ERG | | | | | | | | | | | | | | | | | | | | | | | | | |
| | MAP3K13 | | | | | | | | | | | | | | | | | | | | | | | | | |
| | CTNNB1 | | | | | | | | | | | | | | | | | | | | | | | | | |
| | EGF | | | | | | | | | | | | | | | | | | | | | | | | | |
| | ROS1 | | | | | | | | | | | | | | | | | | | | | | | | | |
| | FGFR1 | | | | | | | | | | | | | | | | | | | | | | | | | |
| | TNKS | | | | | | | | | | | | | | | | | | | | | | | | | |
| | RET | | | | | | | | | | | | | | | | | | | | | | | | | |
| | KMT2A | | | | | | | | | | | | | | | | | | | | | | | | | |
| | SPEN | | | | | | | | | | | | | | | | | | | | | | | | | |
| | POLQ | | | | | | | | | | | | | | | | | | | | | | | | | |
| | APC | | | | | | | | | | | | | | | | | | | | | | | | | |
| | RICTOR | | | | | | | | | | | | | | | | | | | | | | | | | |
| | SYK | | | | | | | | | | | | | | | | | | | | | | | | | |
| | | | | | | | | | | | | | | | | | | | | | | | | | | |
| CNV | CDK4 | | | | | | | | | | | | | | | | | | | | | | | | | |
| | CDKN2A | | | | | | | | | | | | | | | | | | | | | | | | | |
| | CCND3 | | | | | | | | | | | | | | | | | | | | | | | | | |
| | EPHB6 | | | | | | | | | | | | | | | | | | | | | | | | | |
| | FCGR2B | | | | | | | | | | | | | | | | | | | | | | | | | |
| | FGF14 | | | | | | | | | | | | | | | | | | | | | | | | | |
| | FGF4 | | | | | | | | | | | | | | | | | | | | | | | | | |
| | FOXL2 | | | | | | | | | | | | | | | | | | | | | | | | | |
| | GNA11 | | | | | | | | | | | | | | | | | | | | | | | | | |
| | HIST1H3B | | | | | | | | | | | | | | | | | | | | | | | | | |
| | MYCN | | | | | | | | | | | | | | | | | | | | | | | | | |
| | NKX2-1 | | | | | | | | | | | | | | | | | | | | | | | | | |
| | NRG1 | | | | | | | | | | | | | | | | | | | | | | | | | |
| | NTRK3 | | | | | | | | | | | | | | | | | | | | | | | | | |
| | PAK5 | | | | | | | | | | | | | | | | | | | | | | | | | |
| | POLD1 | | | | | | | | | | | | | | | | | | | | | | | | | |
| | PPP1R15A | | | | | | | | | | | | | | | | | | | | | | | | | |
| | SERPINB3 | | | | | | | | | | | | | | | | | | | | | | | | | |
| | SETBP1 | | | | | | | | | | | | | | | | | | | | | | | | | |
| | SMO | | | | | | | | | | | | | | | | | | | | | | | | | |
| | UGT1A1 | | | | | | | | | | | | | | | | | | | | | | | | | |
| | EGFR | | | | | | | | | | | | | | | | | | | | | | | | | |
| | MYC | | | | | | | | | | | | | | | | | | | | | | | | | |
| | APOBEC3B | | | | | | | | | | | | | | | | | | | | | | | | | |
| | FANCF | | | | | | | | | | | | | | | | | | | | | | | | | |
| | RB1 | | | | | | | | | | | | | | | | | | | | | | | | | |
| | SRSF2 | | | | | | | | | | | | | | | | | | | | | | | | | |
| | | | | | | | | | | | | | | | | | | | | | | | | | | |
| | MSI | | MSS | | MSS | | | | MSS | | | | | | | | | | MSS | | | | MSS | | MSS | |
| | TMB | | Low | | Low | | | | Low | | | | | | | | | | Low | | | | Low | | Low | |
EGFR
Del 19 positive
L858R positive
Minor or compound mutation positive
T790M positive
| SNV | |
| --- | --- |
| | |
| | |
| | |
| | |
| | |
Missense variant
Upstream gene variant
Stop gained
| | |
| --- | --- |
| CNV | |
| | |
| | |
| | |
Deletion
Amplification
